# Concurrent Breast and Colon Cancer: Small Cohort Analysis

**DOI:** 10.7759/cureus.107999

**Published:** 2026-04-29

**Authors:** Qasif Qavi, KM Saiful Islam, Zeeshan Hashmi, Rizwan Ahmed, Jafer Hamdani, Noreen Rasheed, Fouzia Rani, Dennis Wayne Chicken, Philip Idaewor, Abdalla Saad Abdalla Al-Zawi

**Affiliations:** 1 Surgery, Basildon and Thurrock University Hospital, Basildon, GBR; 2 Surgery, King’s College Hospital NHS Foundation Trust, London, GBR; 3 Cardiology, King’s College Hospital NHS Foundation Trust, London, GBR; 4 Surgery, Mersey and West Lancashire Teaching Hospitals NHS Trust, Knowsley, GBR; 5 Surgery, Princess Royal University Hospital, Orpington, GBR; 6 General Surgery, Basildon and Thurrock University Hospital, Basildon, GBR; 7 Radiology, Basildon and Thurrock University Hospital, Basildon, GBR; 8 General Surgery, Mid and South Essex NHS Foundation Trust, Basildon, GBR; 9 Histopathology/Cellular Pathology, Mid and South Essex NHS Foundation Trust, Basildon, GBR; 10 Histopathology/Cellular Pathology, Basildon and Thurrock University Hospital, Basildon, GBR; 11 General and Breast Surgery, Mid and South Essex NHS Foundation Trust, Basildon, GBR; 12 General and Breast Surgery, Basildon and Thurrock University Hospital, Basildon, GBR; 13 General and Breast Surgery, Anglia Ruskin University, Chelmsford, GBR

**Keywords:** breast cancer, colorectal cancer, metachronous cancer, multiple primary malignant neoplasms, synchronous cancer

## Abstract

Introduction

Multiple primary malignant neoplasms (MPMNs) are increasingly recognized due to improved diagnostics and prolonged survival. Predisposing factors include genetic mutations, lifestyle, and environmental exposures. This study analyzes the clinic-pathological characteristics of female patients diagnosed with synchronous or metachronous bowel and breast cancers to highlight diagnostic challenges and management strategies.

Material and methods

A retrospective review was conducted on 12 female patients treated for both malignancies between 2015 and 2025. The study analyzed demographic data, clinical presentation, radiological findings, histopathological features, genetic associations, and therapeutic interventions.

Results

The mean patient age was 70 years (range: 56-84). Seven cases (58%, n=7) were synchronous. In 33% (n=4) of patients, the breast primary was detected incidentally during colon cancer staging. Histopathologically, breast tumours were predominantly invasive carcinoma of no special type. Colonic tumours were mostly adenocarcinomas (91%, n=10), though rare co-pathologies, including a neuroendocrine tumour and anal squamous cell carcinoma, were observed. Curative surgery was performed in 75% (n=8) of breast and 91% (n=10) of colonic cases. The overall mortality rate was 8.3% (n=2).

Conclusion

MPMNs are becoming more frequent in both elderly and younger demographics. The concurrence of breast and colonic cancers - often metachronous - can arise without identifiable risk factors. These complex cases require an individualized, multidisciplinary approach to ensure timely diagnosis and optimal outcomes.

## Introduction

The co-occurrence of bowel and breast cancer, whether synchronously (those detected simultaneously or within six months of each other) or metachronously (identified more than six months after the initial diagnosis), presents a complex clinical challenge. Multiple primary malignant neoplasms (MPMNs) - defined as two or more distinct primary cancers - are increasingly recognized in clinical practice [1,2,3]. Although breast and colorectal cancers are individually among the most common malignancies, their synchronous occurrence is considered rare [4,5]. This case series highlights the varied presentations and characteristics of patients with both malignancies, supported by a review of the literature on synchronous and metachronous presentations [6]. Understanding these cases in the context of current research is essential for informing clinical management and guiding future investigation.

## Materials and methods

This retrospective cohort study examined 12 cases of patients diagnosed with synchronous or metachronous primary breast and colonic cancers at the Mid & South Essex NHS Foundation Trust between January 2015 and December 2025. Data were identified through a comprehensive search of the Trust’s electronic health records and multidisciplinary team (MDT) databases, using a six-month threshold to differentiate between synchronous (diagnosed within six months) and metachronous (diagnosed >6 months apart) presentations. For each case, a standardized data extraction protocol was utilized to collect demographic information (age and gender), clinical presentation, and diagnostic workup, including mammography, ultrasonography, computed tomography (CT), and endoscopic findings. Detailed histopathological and immunohistochemical profiles were reviewed to confirm distinct primary origins and exclude metastatic disease, specifically evaluating tumor grade, TNM staging, and molecular markers (ER, PR, HER2, and MSI status). Management strategies were further analyzed, encompassing surgical interventions, chemotherapy, radiotherapy, and endocrine therapies, with all data anonymized to ensure patient confidentiality in accordance with institutional ethical guidelines.

## Results

This case series of 12 patients highlights the diverse clinical presentations observed in individuals diagnosed with both bowel and breast cancer. All patients were female, with ages at first diagnosis ranging from 56 to 84 years (mean: 70 years). Two patients (Patients 6 and 7) were younger than 60, and two (Patients 2 and 5) were older than 80 (Table 1).

**Table 1:** Clinico-pathological features and management of the cases IHC: Immunohistochemistry; IBC, NST: Invasive breast carcinoma, of no special type; ER: Oestrogen receptor; PR: Progesterone receptor; HER2: Human epidermal growth factor receptor; Mx: Mastectomy; ANC: Axillary node clearance; SLNB: Sentinel lymph node biopsy; BCS: Breast conservation surgery; MD: Moderately differentiated; PD: Poorly differentiated; ET: Endocrine treatment.

No.	Age	Gender	Breast cancer				Bowel Cancer		Presentation
			Histology	Grade	IHC	Treatment	Histology	Treatment	
1	68	F	IBC,NST	3	ER/PR +ve,HER2 -Low	Mx +ANC	MD adenocarcinoma	Right hemicolectomy	Synchronous
2	84	F	IBC,NST	2	ER/PR +ve,HER2 -ve	NONE	PD adenocarcinoma +NET ileum	Right Hemicolectomy	Synchronous
3	70	F	IBC,NST	2	ER/PR +ve,HER2 -Low	BCS+ANC	MD Adenocarcinoma	Right Hemicolectomy	Metachronous-
4	77	F	IBC,NST	3	ER/PR -ve,HER2 Low	BCS+ANC	WD Adenocarcinoma +NET	None	Synchronous
5	81	F	IBC,NST	2	ER+ve/PR -ve,HER2 +ve	BCS+SLNB	MD Adenocarcinoma	Right Hemicolectomy	Synchronous
6	56	F	IBC,NST	2	ER/PR +ve,HER2 -Low	BCS+SLNB	MD Adenocarcinoma	Low Anterior Resection	Synchronous
7	58	F	IBC,NST	3	ER/PR +ve,HER2 -ve	BCS+SLNB	MD Adenocarcinoma	Right Hemicolectomy	Metachronous
8	66	F	IBC,NST	1	ER/PR +ve,HER2 -ve	BCS+ANC	MD Adenocarcinoma	Low Anterior Resection	Metachronous
9	72	F	IBC,NST	2	ER/PR +ve,HER2 –Low	NONE	MD Adenocarcinoma	Right Hemicolectomy-Lap	Metachronous
10	70	F	ILC	2	ER/PR +ve,HER2 -ve	BCS	PD Adenocarcinoma	Right Hemicolectomy	Synchronous
11	74	F	IBC,NST	3	ER/PR +ve,HER2 +ve	NONE	SCC	None-CTH only	Synchronous
12	67	F	IBC,NST	2	ER/PR+ve, HER2+ve	None	MD Adenocarcinoma	Right Hemicolectomy	Metachronous

**Figure 1:**
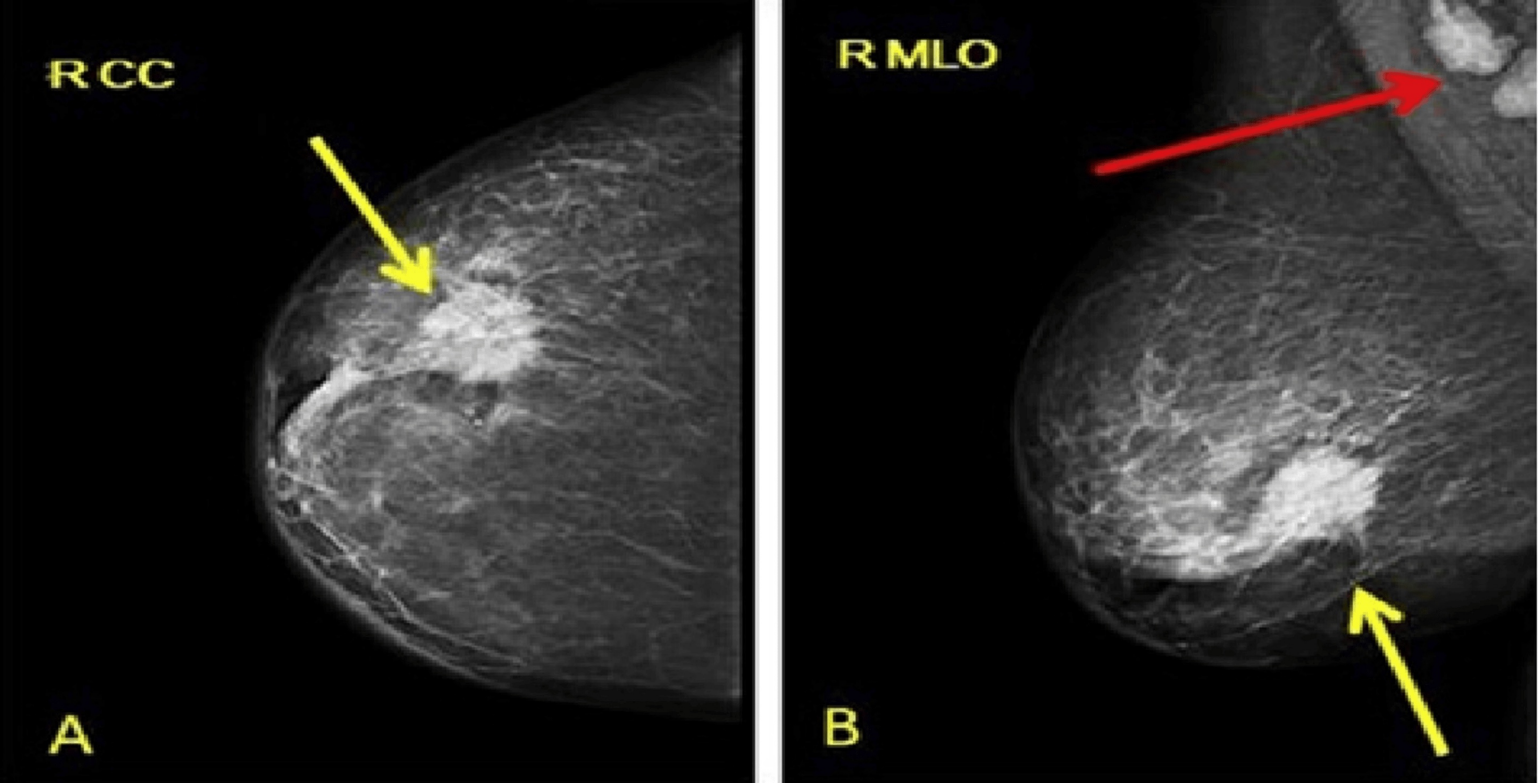
(Case 1) Right mammogram: (A) CC view, (B) MLO view reveals a highly suspicious 32 mm M5 lesion located in the lower outer quadrant of the right breast (Yellow arrows), along with A4-grade prominent right axillary lymphadenopathy (Red arrow). CC: Craniocaudal; MLO: Mediolateral oblique

**Figure 2:**
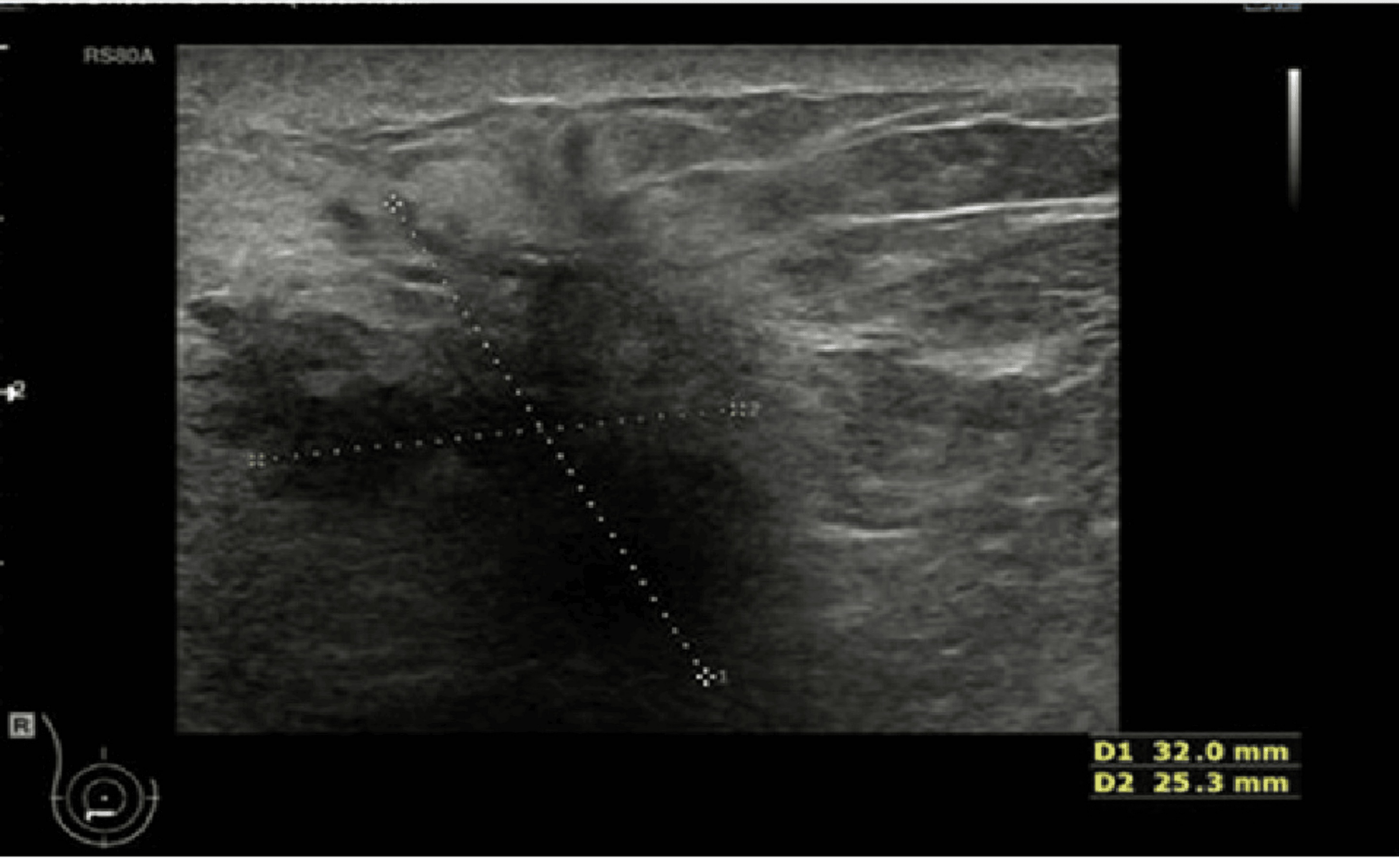
(Case 1) Right breast ultrasound scan reveals a highly suspicious 32 mm U5 lesion located in the lower outer quadrant of the right breast.

**Figure 3:**
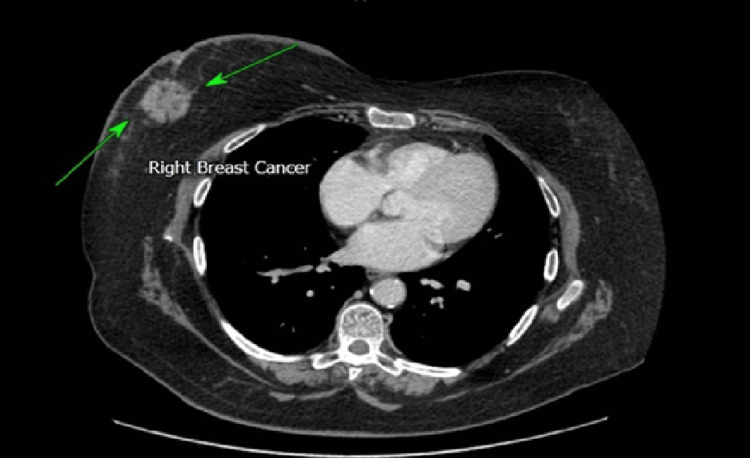
(Case 1) Axial plane contrast-enhanced CT image shows evidence of right breast carcinoma (Green arrows) associated with overlying skin thickening.

**Figure 4:**
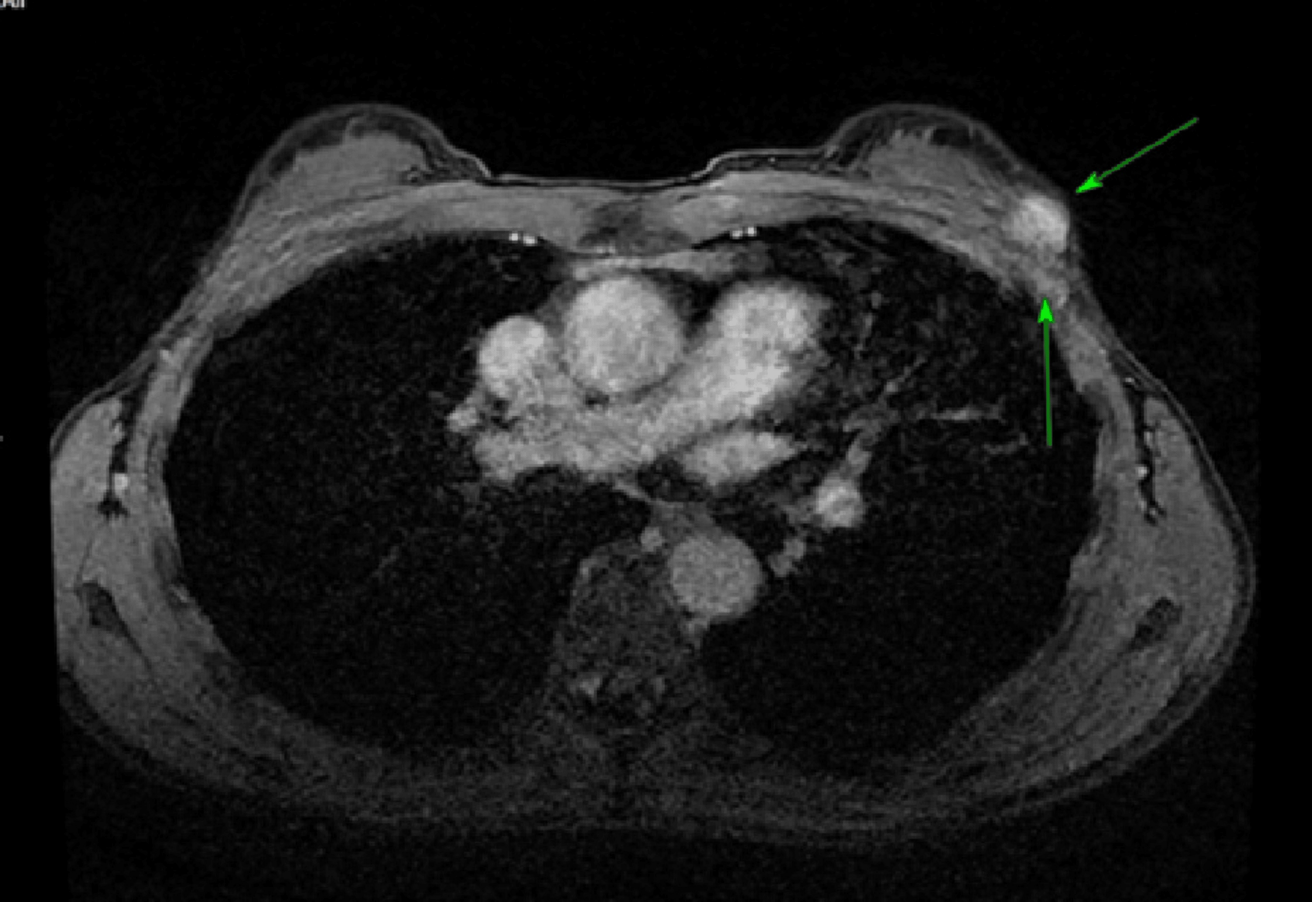
(Case 6) Axial plane MRI breasts shows left breast carcinoma.

**Figure 5:**
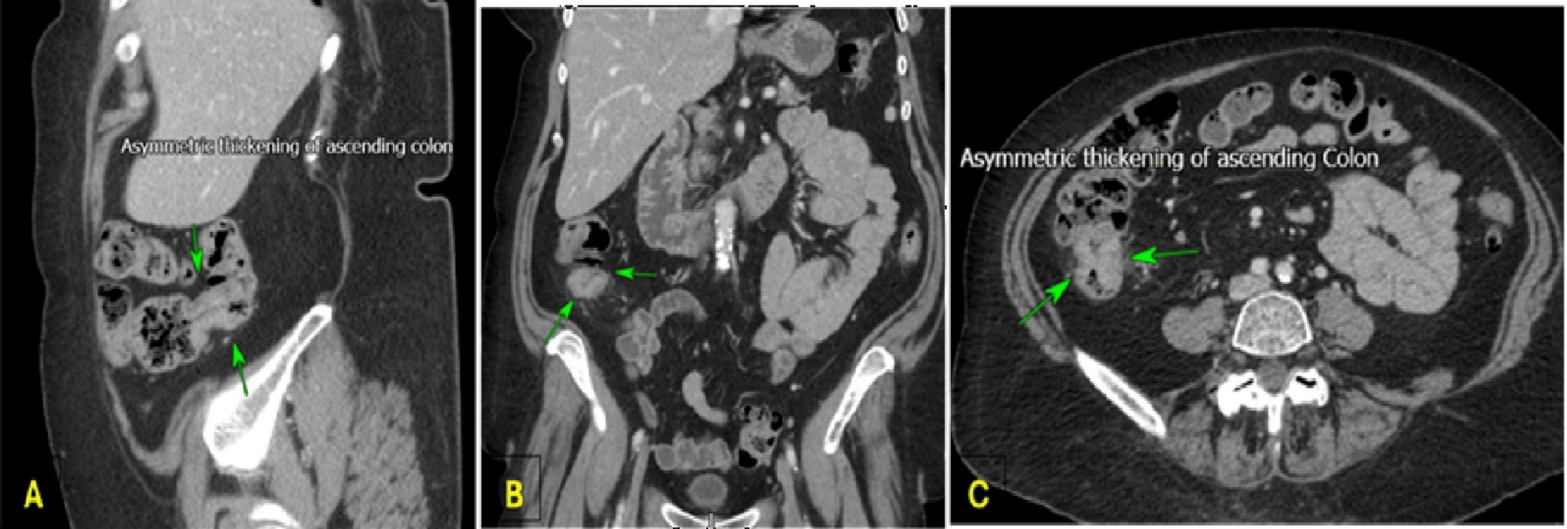
(Case 1) (A) Sagittal view, (B) Coronal view, (C) Axial view: Abdominal contrast-enhanced computerised tomography scan reveals asymmetric thickening of the ascending colon (Green arrows).

**Figure 6:**
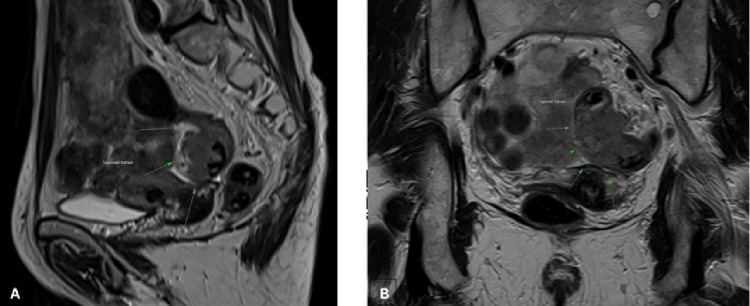
(Case 6) (A) Sagittal view, (B) Coronal view: MRI pelvis showing 4 cm sigmoid colon tumour (Green arrows).

**Figure 7:**
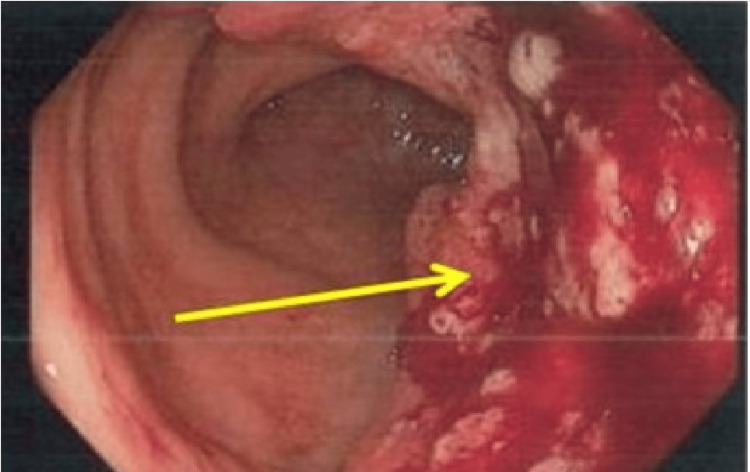
(Case 6) Colonoscopy view showing suspicious polypoidal tumour extending from the recto-sigmoid junction to sigmoid colon (Yellow arrow).

**Figure 8:**
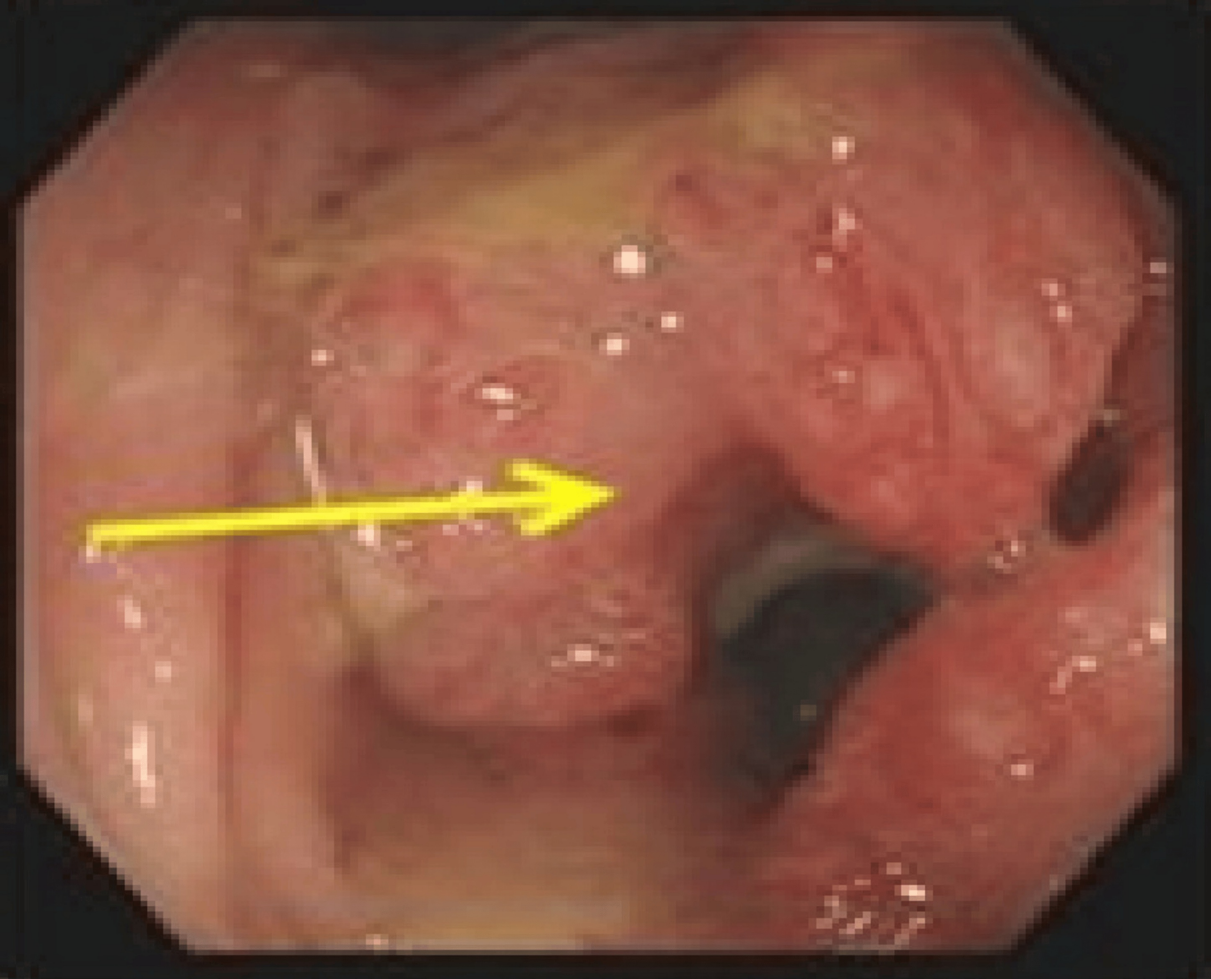
(Case 5) Colonoscopy view revealing a tumour covers all the lumen of the caecum.

The cohort included seven synchronous cases - Patients 1, 2, 4, 5, 6, 10, and 11 - representing 58% (n=7) of the series. In Patients 5, 6, and 9, the initial presentation was a breast lump, evaluated with mammography (Figure 1) and breast ultrasound (Figure 2). In Patients 1, 2, 5, and 10 (33%,n=4), the breast primary was detected incidentally on CT scans performed for bowel symptoms investigation or colon cancer staging (Figures 3-5). Additional bowel tumour evaluation with abdominal MRI was also offered for some cases (Figure 6). Once bowel pathology was suspected, colonoscopy was performed, and tissue diagnosis was obtained (Figures 7-8).

Regarding the anatomical distribution of colonic tumours, four were located in the caecum, four in the ascending colon, two in the sigmoid colon, one in the splenic flexure, and one in the anal canal.

**Figure 9:**
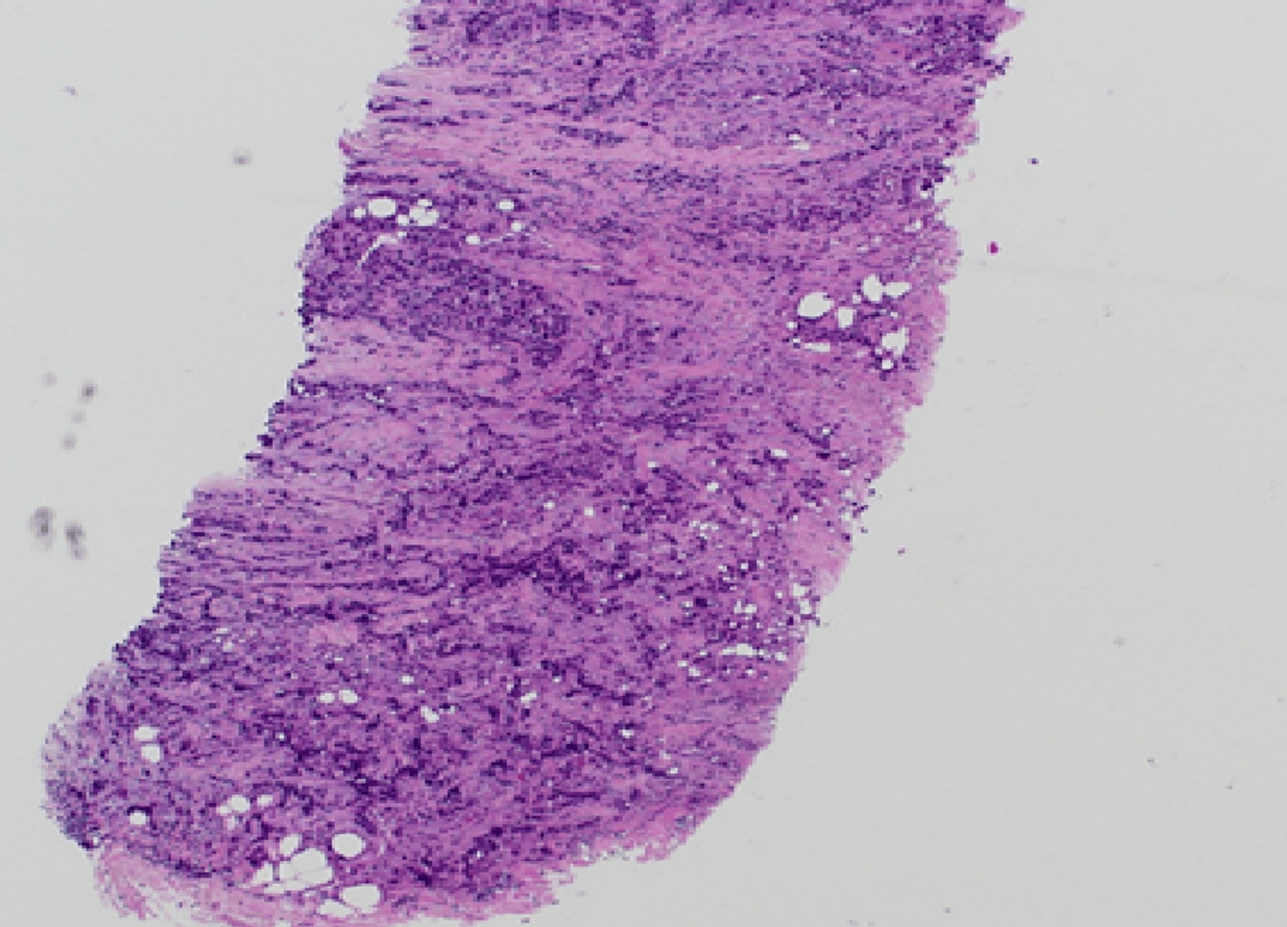
(Case 1) H&E 40x. Breast core biopsy containing invasive carcinoma NST Grade 3. NST: no special type

**Figure 10:**
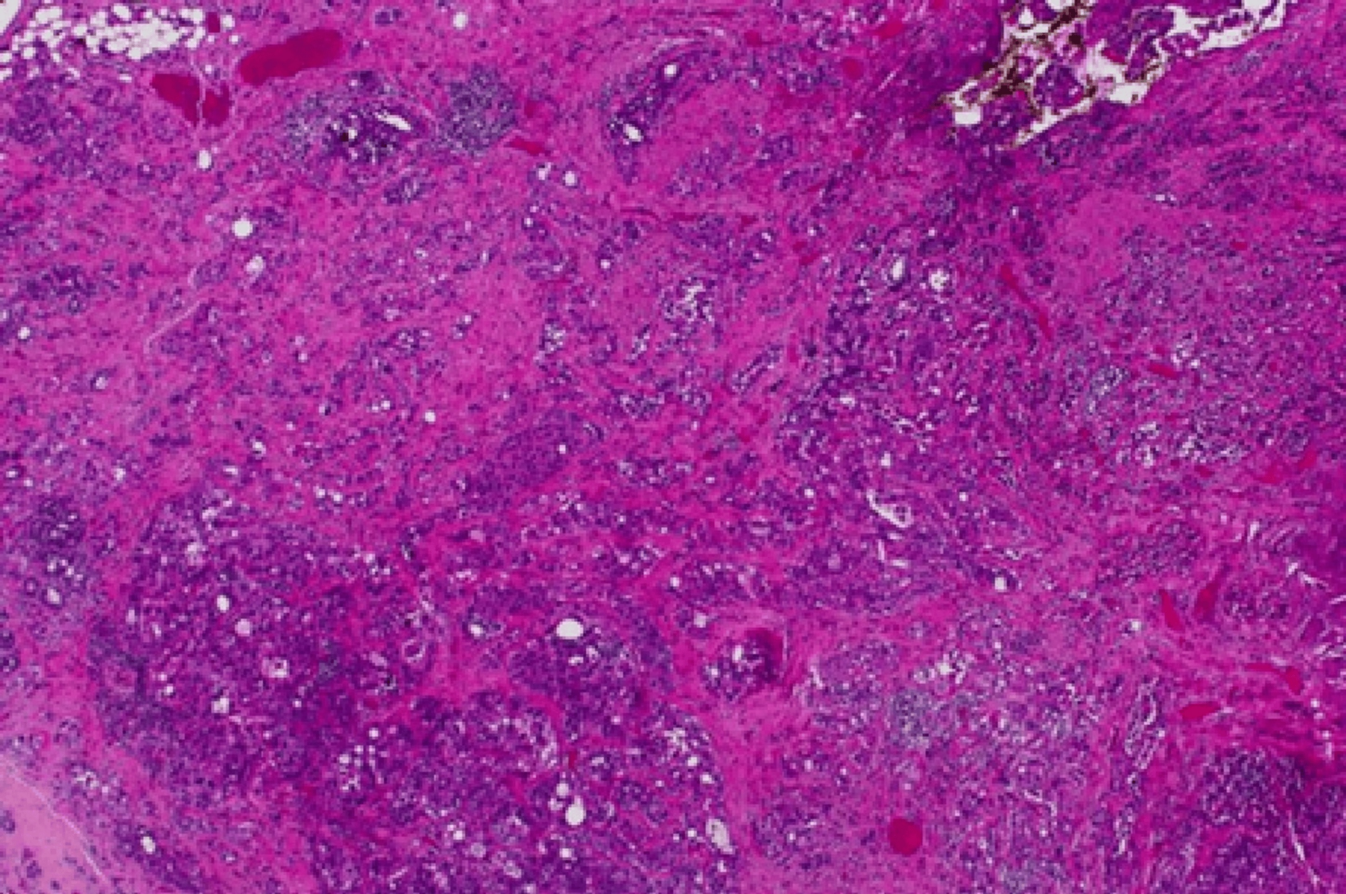
(Case 6) H&E 40x. Left breast WLE containing invasive carcinoma NST Grade 2. WLE: Wide local excision; NST: no special type

**Figure 11:**
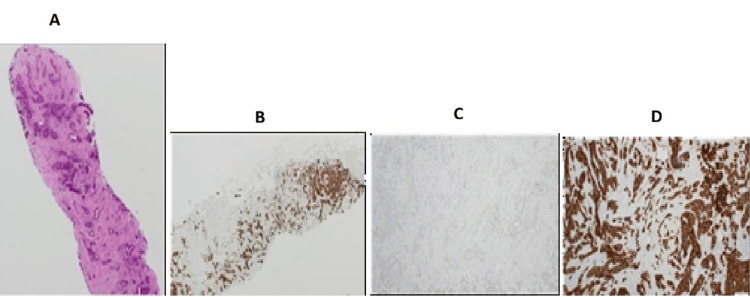
(Case 5) Breast core biopsy infiltrated by invasive carcinoma NST (formerly called invasive ductal carcinoma) Grade 2. (A) H&E-40x, (B) ER positive (All red 8/8), (C) PR negative (All red 0/8), (D) Her-2 Positive

Histopathological examination revealed invasive breast carcinoma of no special type (IBC, NST) - formerly termed invasive ductal carcinoma - in all but one case (Figures 9-11). The exception, Patient 10, had invasive lobular carcinoma (ILC). Eight patients had ductal carcinoma in situ (DCIS) alongside invasive breast carcinoma of no special type, while the patient with ILC had pleomorphic lobular carcinoma in situ (P-LCIS) of the breast.

**Figure 12:**
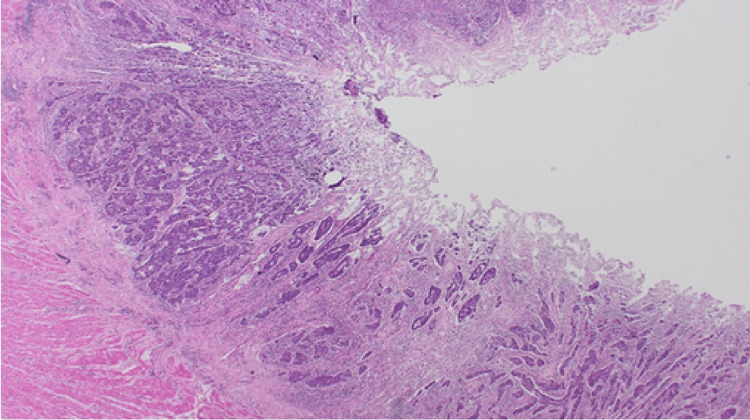
(Case 1) H&E 20x. Colonic resection containing adenocarcinoma.

**Figure 13:**
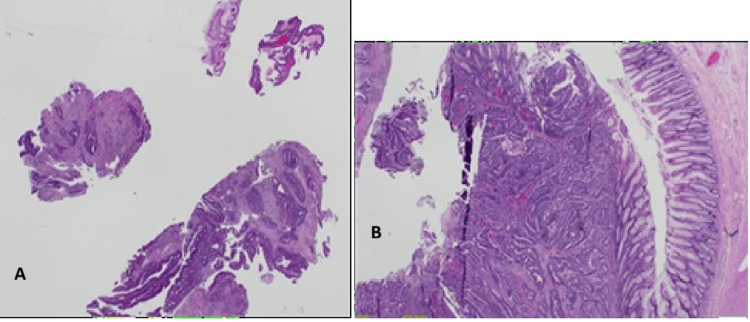
(A) H&E 20x Colonic biopsy. (B) H&E 20x rectosigmoid resection colonic biopsy and resection containing moderately differentiated adenocarcinoma from the rectosigmoid. This tumour was MMR proficient. MMR: Mismatch repair

**Figure 14:**
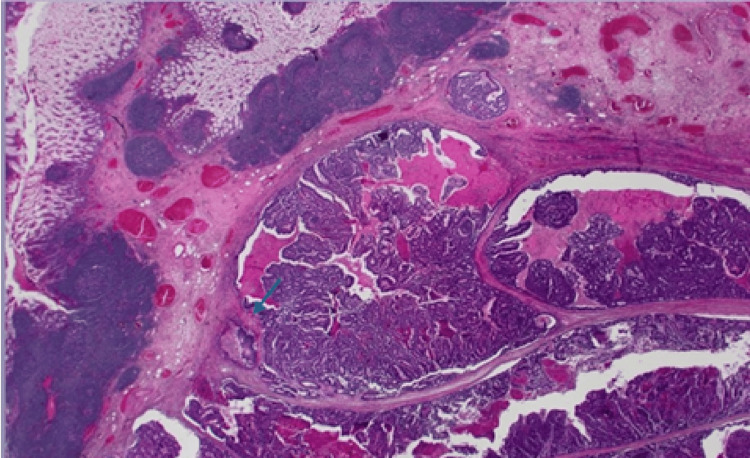
(Case 5) H&E Colonic resection specimen containing moderately differentiated adenocarcinoma. Mag 12.5x (This was diagnosed a year earlier to the metastasis to the liver and morphologically, this tumour and the liver metastasis appear similar).

**Figure 15:**
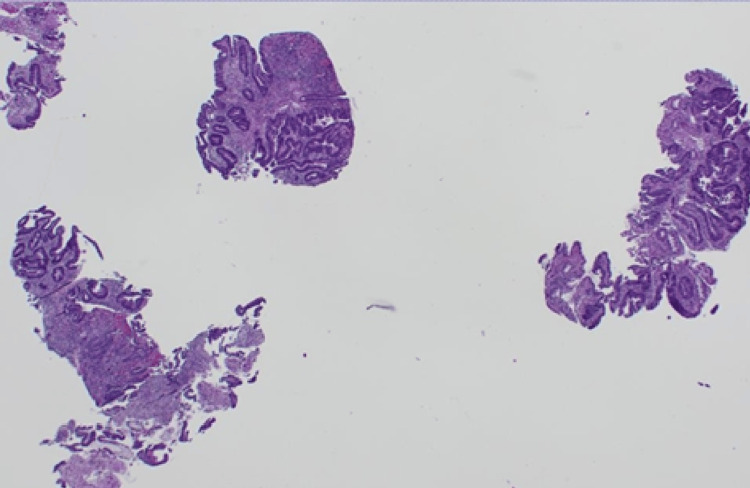
(Case 8) H&E 20x. Polyps from rectum. It showed high grade dysplasia and adenocarcinoma.

Among the cohort, primary colonic tumours were predominantly adenocarcinomas, accounting for 91%(n=10) (Figures 12-15), with one case coexisting with a neuroendocrine tumour of the ileum. Only one patient was diagnosed with squamous cell carcinoma of the anal canal.

The “Grade of Breast Cancer” column in Table 1 provides further details on tumour differentiation: grade 2 was observed in seven cases (58%, n=7), and grade 3 in four cases (33%, n=4).

Invasive breast carcinoma of no special type (IBC, NST) - historically referred to as invasive ductal carcinoma - represented the predominant histological subtype in this cohort, being identified in all cases except one (Figures 9-11). The sole exception, Patient 10, demonstrated invasive lobular carcinoma (ILC), a distinct morphological entity characterized by its unique growth pattern and clinical behaviour. Notably, eight patients exhibited coexistent ductal carcinoma in situ (DCIS) in association with IBC, underscoring the frequent synchronous presentation of in situ and invasive components within the same breast. In contrast, the patient diagnosed with ILC harboured pleomorphic lobular carcinoma in situ (P-LCIS), a histologically aggressive variant of lobular neoplasia, further highlighting the heterogeneity of precursor lesions accompanying invasive disease. These findings emphasize the spectrum of pathological alterations encountered in invasive breast cancer and their potential implications for diagnosis, treatment planning, and prognostic assessment.

The colonic adenocarcinoma differentiation also varied, with moderate differentiation in seven cases (58%, n=7), poor differentiation in two cases (16%, n=2), and well differentiation in one case. A statistical comparison of the moderate differentiation category between breast and colonic cancer showed no significant association (OR = 1.25; 95% CI: -0.25 to 0.27; P = 1.0).

**Figure 16:**
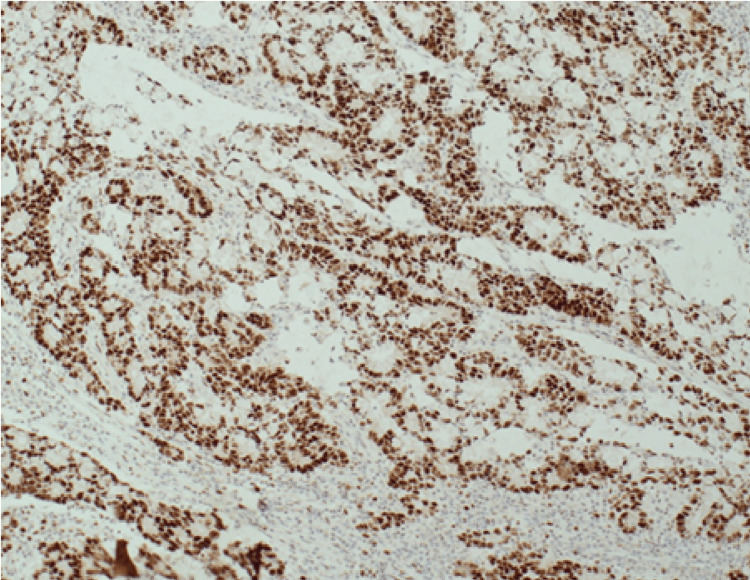
(Case 1) Colonic tumour from right hemicolectomy strongly positive for MSH6 (MutS homolog 6)x10.

In one case, the diagnosis included synchronous invasive breast carcinoma with ductal carcinoma in situ (DCIS) and renal cell carcinoma, along with metachronous colonic cancer. The cohort also included a case of multiple primary malignant neoplasms (MPMNs) involving breast, colonic, and ovarian primaries. One case has developed liver metastasis from the colon origin after receiving treatment for both breast and colonic cancers (Figure 16).

Genetic mutations predisposing to the development of both breast and colonic cancers were identified in 50% of the cohort. Four cases (33%, n=4) were confirmed to have Lynch syndrome with an MSH6 mutation (MutS homolog 6), one case had a BRCA1 mutation, and one case had a KRAS G12V mutation. Figure 16 (Case no. 1) shows strongly positive colonic tumour cells for MSH6, supporting its role in the diagnosis of Lynch syndrome.

**Figure 17:**
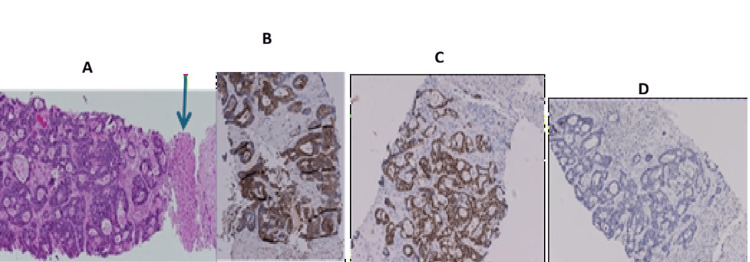
(Case 5) Liver core biopsy containing metastatic colonic adenocarcinoma, mag 40x. A small segment of normal liver tissue is seen in the right side of the micrograph (see blue arrow in A). The immunoprofile is consistent with metastatic adenocarcinoma of colonic origin. This tumour is MMR proficient. (A) H&E, (B) CK20 positive, (C) CDX2 positive, (D) GATA3 negative MMR: Mismatch repair

In Case no. 8, the analysis of a rectal polyp showed high-grade dysplasia and adenocarcinoma. The tumour was MMR proficient, Her-2 negative, and no clinically actionable variants were detected in BRAF, KRAS and NRAS genes, consistent with wild-type RAS/BRAF. This tumour, therefore, may not respond to anti-EGFR antibody therapy. Also, no clinically actionable variants were detected in the regions analysed in the PIK3CA, ERB2 and MET genes. Also, microsatellite instability (MSI) was not detected (microsatellite stable tumour) (Figure 17).

Two cases underwent mastectomy, and seven (60%) received breast-conserving surgery for breast cancer, while three cases were treated with primary endocrine therapy. A total of four patients underwent axillary clearance due to nodal disease.

Right hemicolectomy for colonic tumours was performed in nine cases (75%), reflecting the predominance of right-sided tumour location in the cohort. Two cases underwent anterior resection, and one case of anal tumour was treated with chemo-radiation therapy.

## Discussion

The occurrence of multiple primary malignant neoplasms (MPMNs), including the combination of breast and colorectal cancer, is increasingly recognized [1, 2]. Both cancers are among the most frequently diagnosed malignancies worldwide [7]. According to the GLOBOCAN 2024 report (World Health Organization Global Cancer Observatory), the most frequently diagnosed cancer worldwide was lung cancer (12.4%), followed by female breast cancer (11.6%), colorectal cancer (9.6%), prostate cancer (7.3%), and stomach cancer (4.9%). The leading cause of cancer-related mortality was lung cancer (18.7%), followed by colorectal (9.3%), liver (7.8%), female breast (6.9%), and stomach cancer (6.8%). Breast cancer remains the most common malignancy in females, while lung cancer is the most frequent cancer in males [8].

The demographics of our case series show that all patients were female, which may be consistent with the higher incidence of breast cancer in women [2,5,9]. However, the incidence rate of colorectal cancer is generally higher in males [10]. The ages at diagnosis varied; however, in a study published by Silverstein et al. in 2024, fifty-one patients younger than 60 years were enrolled. All had been diagnosed with breast and colonic cancers, often without known risk factors, and only 25% had a confirmed genetic predisposition. Alcohol consumption and a sedentary lifestyle were also considered potential risk factors [11].

In our cohort, some patients were younger than 60 (36%, n=4). This highlights the fact that the incidence of breast and colorectal cancer (CRC) in younger-than-average-age patients may be increasing.

Many researchers have mentioned the coexistence of common extrinsic and genetic predisposing factors related to the concurrence of both breast and colonic cancers [3]. BRCA1, BRCA2 and CHEK2 mutations are well known to be associated with breast cancer. They also have a moderately increased risk of colorectal cancer [12].

Mutation carriers in the genes MLH1 (MutL homolog 1), MSH2, MSH6 (MutS homolog 6), PMS2 (Post-Meiotic Segregation increased 2), and EPCAM (Epithelial Cell Adhesion Molecule) are associated with Lynch syndrome - formerly known as hereditary nonpolyposis colorectal cancer (HNPCC) - and have an increased risk of developing colorectal, endometrial, ovarian, small bowel, pancreatic, prostate, urothelial, and breast cancers [2, 13, 14].

Synchronous diagnoses - defined as the detection of two primary cancers within a six-month period - are rare, with an incidence of 3.85% [15]. The second primary cancer is often identified during the staging workup for the initially diagnosed malignancy [16, 17]. For example, in Patient 2, the breast tumour was incidentally discovered on a CT scan performed for colon cancer staging.

Metachronous diagnoses - defined as the detection of one cancer more than six months after another - appear to be more common [3,16]. Notably, a study focusing on patients under 60 years of age found that 86.3% of colorectal cancer cases were metachronous, following an initial breast cancer diagnosis [11]. In our older patient cohort (mean age 74 years), eight cases (72%) were metachronous.

Not uncommonly, clinicians face a significant diagnostic challenge when managing multiple primary cancers [18]. The cases of patients 3, 9, and 12 underscore the importance of distinguishing a new primary cancer from a recurrence. In our cohort, nine patients were diagnosed with invasive breast carcinoma accompanied by ductal carcinoma in situ (DCIS); in four of these cases, the non-invasive component showed a high nuclear grade. This further highlights the variety of breast cancer subtypes encountered in these patients [1]. Patient 8’s case - involving invasive breast carcinoma with ductal carcinoma in situ (DCIS) and synchronous renal cell carcinoma - highlights the possibility of breast cancer co-occurring with more than one additional primary malignancy [16].

The management of these complex cases invariably requires a multidisciplinary approach, as demonstrated by the treatment strategies used for several patients [15]. The decision-making process involves specialists from multiple disciplines to determine the optimal sequence and combination of surgical, chemotherapeutic, and radiotherapeutic interventions. Breast cancer treatment employed diverse surgical approaches, including mastectomy, breast-conserving surgery (BCS), and, when indicated, sentinel lymph node biopsy (SLNB) or axillary clearance, reflecting individualized treatment plans based on tumour characteristics and patient factors.

The high rate of BCS indicates early disease detection during colorectal disease diagnosis and staging.

Surgical management of colorectal malignancies included procedures such as right hemicolectomy, extended right hemicolectomy, laparoscopic resections, and low anterior rectal resection, tailored to the tumour’s location and extent [4].

While the scope of this case series does not allow for a detailed analysis of risk factors, the literature suggests potential roles for genetic predispositions and lifestyle factors in the development of multiple primary cancers [2]. Further research is needed to explore these associations in greater depth.

## Conclusions

This case series, integrated with a review of the current literature, provides an overview of the clinical presentations of synchronous and metachronous bowel and breast cancers. The rarity of synchronous cases and the higher frequency of metachronous diagnoses align with existing findings. These cases, together with the broader body of research, underscore the importance of thorough investigation, multidisciplinary management, and long-term surveillance for patients with a history of either breast or colorectal cancer.
